# Conformational switches and redox properties of the colon cancer‐associated human lectin ZG16

**DOI:** 10.1111/febs.16044

**Published:** 2021-06-15

**Authors:** Gabriel Javitt, Alisa Kinzel, Nava Reznik, Deborah Fass

**Affiliations:** ^1^ Department of Chemical and Structural Biology Weizmann Institute of Science Rehovot Israel

**Keywords:** *cis* peptide, disulfide, lectin, QSOX1, X‐ray crystallography

## Abstract

Zymogen granule membrane protein 16 (ZG16) is produced in organs that secrete large quantities of enzymes and other proteins into the digestive tract. ZG16 binds microbial pathogens, and lower ZG16 expression levels correlate with colorectal cancer, but the physiological function of the protein is poorly understood. One prominent attribute of ZG16 is its ability to bind glycans, but other aspects of the protein may also contribute to activity. An intriguing feature of ZG16 is a CXXC motif at the carboxy terminus. Here, we describe crystal structures and biochemical studies showing that the CXXC motif is on a flexible tail, where it contributes little to structure or stability but is available to engage in redox reactions. Specifically, we demonstrate that the ZG16 cysteine thiols can be oxidized to a disulfide by quiescin sulfhydryl oxidase 1, which is a sulfhydryl oxidase present together with ZG16 in the Golgi apparatus and in mucus, as well as by protein disulfide isomerase. ZG16 crystal structures also draw attention to a nonproline *cis* peptide bond that can isomerize within the protein and to the mobility of glycine‐rich loops in the glycan‐binding site. An understanding of the properties of the ZG16 CXXC motif and the discovery of internal conformational switches extend existing knowledge relating to the glycan‐binding activity of the protein.

## Graphical abstract

Zymogen granule membrane protein 16 (ZG16) is an animal lectin present in the intestine. Here, we show that ZG16 has a redox‐active CXXC disulfide in a non‐canonical structural context: on a flexible, carboxy‐terminal tail. Additional conformational heterogeneity is seen in the glycan‐binding region of ZG16, including a rare *cis‐trans* isomerization of a peptide bond that does not precede a proline.

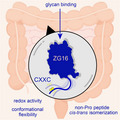

**Abbreviations**
DTT: dithiothreitol
ER: endoplasmic reticulum
mal‐PEG: maleimide‐functionalized polyethylene glycol
MBP: maltose binding protein
non‐Pro: nonproline
ox: oxidized
PBS: phosphate‐buffered saline
PDI: protein disulfide isomerase
PEG: polyethylene glycol
QSOX1: quiescin sulfhydryl oxidase 1
red: reduced
TEV: tobacco etch virus
Trx: thioredoxin
ZG16: zymogen granule membrane protein 16

## Introduction

Zymogen granule membrane protein 16 (ZG16) is a mammalian lectin‐like protein produced at high levels in the colon and also found in the pancreas, liver, and other tissues [1, 2, 3]. ZG16 downregulation is associated with ulcerative colitis and colon cancer [4, 5, 6, 7, 8, 9]. Intracellularly, ZG16 is localized to the Golgi apparatus and zymogen granule membranes [10, 11]. Extracellularly, it is found in the mucus‐associated proteome [12]. The function of ZG16 is not yet known, but it may aid in the packaging of glycoproteins into secretory granules [13] and has been shown to decrease the penetration of bacteria into the mucus that coats the colon epithelium [14]. The ability of ZG16 to bind diverse glycans [15, 16, 17] is consistent with a putative role in innate immunity or in handling secretory cargo or both.

**Fig. 1:**
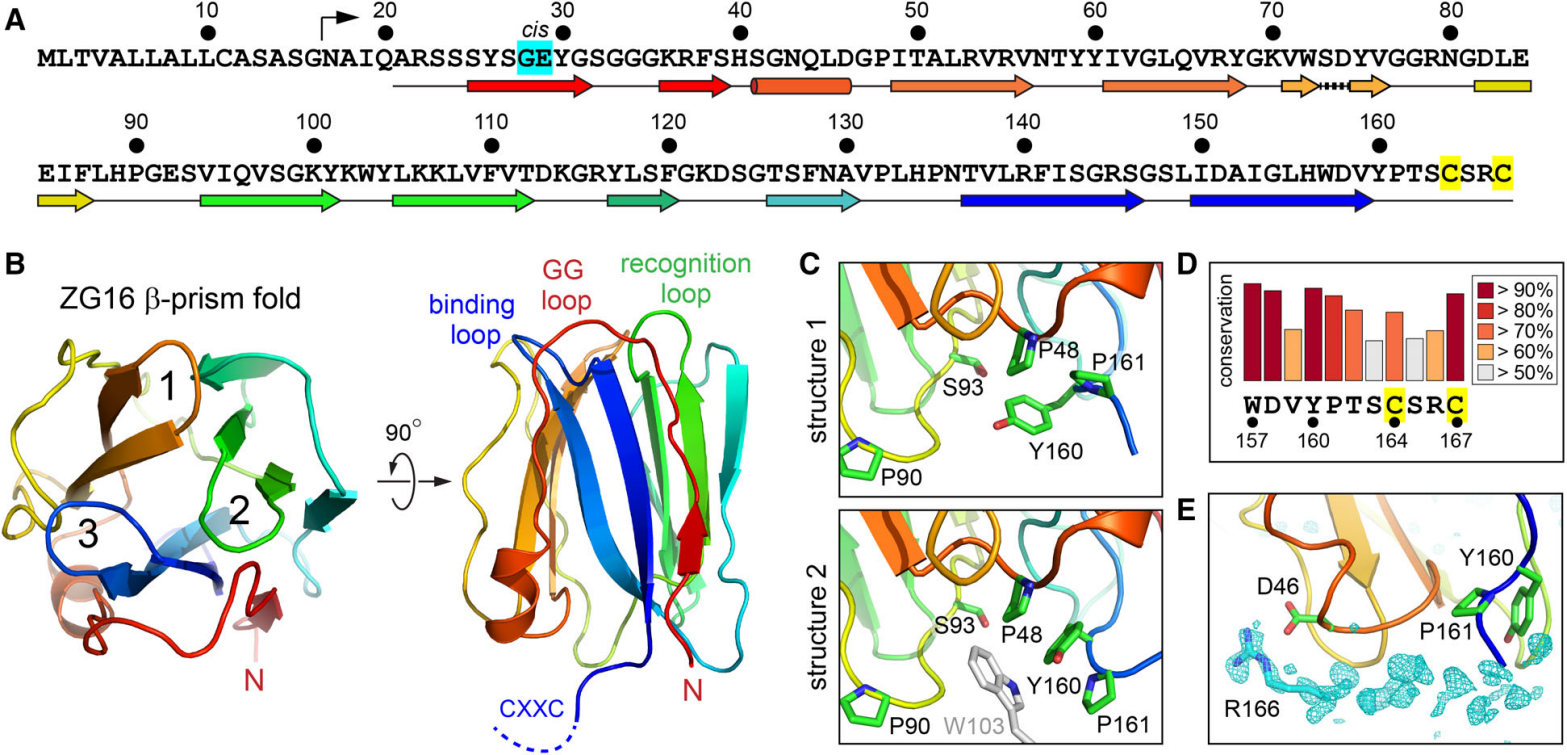
Structure of ZG16 highlighting the flexible carboxy terminus. (A) Amino acid sequence of human ZG16. The bent arrow above the sequence indicates the predicted start of the mature protein after signal peptide cleavage. Secondary structure assignments are shown below the sequence and colored to correspond to panel B. The segment from residue 71–76 is presented with a dashed line because the side chain of Ser73, rather than the backbone, hydrogen bonds to the adjacent β‐hairpin. The residues flanking the *cis* peptide bond present in some of the ZG16 structures are highlighted in cyan and the cysteines in yellow. (B) Ribbon diagram of ZG16 in two orientations, made using structure 1 and colored from red (N terminus) to blue (C terminus). Numbers indicate the β hairpins. A dashed line representing the CXXC motif was added for the purpose of illustration. Loops forming the glycan‐binding site are labeled according to [15]. (C) Comparison of two ZG16 structures illustrates the flexibility of the region containing Tyr160 and Pro161. In structure 2, these residues assume a different conformation to accommodate a crystal packing interaction with Trp103 (gray). (D) Conservation of amino acids in the carboxy‐terminal tail of ZG16. (E) A Fo‐Fc map for chain B of structure 3 displayed at 3σ extending to 6 Å from the protein. The Arg166 side chain was not used to calculate the map but was subsequently placed into the appropriate density. Structure figures were made using pymol.

Previous structural studies focused on the carbohydrate binding capabilities of ZG16 [15, 16], but the redox activity of the protein has not yet been addressed. ZG16 has a CXXC (Cys‐Xxx‐Xxx‐Cys) amino acid sequence (Fig. 1A), which is CSRC in human ZG16 (UniProt O60844). A CXXC motif is a feature shared by thiol‐disulfide oxidoreductases of the thioredoxin (Trx) superfamily [18] and sulfhydryl oxidases such as ERV/ALR enzymes [19] and Ero1 [20], all of which display this motif at the amino terminus of a helix. Precluding a similar structural context in ZG16, the CXXC is the final four amino acids at the carboxy terminus of the protein. No information was available about the ZG16 CXXC from previous crystal structures since the final eight carboxy‐terminal residues were omitted from the crystallized variant [21].

Here we report the crystallization and analysis of ZG16 containing the CXXC motif. In efforts to resolve the structure of the CXXC region, we analyzed multiple high‐resolution crystal structures, which fortuitously provided information on conformational flexibility within the folded protein domain. In addition to the structural analysis of ZG16 including the CXXC, we also addressed ZG16 redox properties with respect to catalysts of disulfide bond formation that function in the endoplasmic reticulum (ER), Golgi apparatus, and perhaps within the intestinal mucus hydrogel.

## Results

### Preparation and crystallization of ZG16

Human ZG16, which has no canonical N‐linked glycosylation sites, was produced in *Escherichia coli* fused carboxy‐terminally to maltose binding protein (MBP), similarly to a previous expression method for this protein [21]. In our work, the ZG16 sequence spanned residues 21–167, rather than just the core lectin domain (residue 21–159) (Fig. 1A). A His_6_ tag and a tobacco etch virus (TEV) protease cleavage site were introduced between the MBP and ZG16. However, we found that ZG16 precipitated upon TEV cleavage. Precipitation could be minimized by increasing the ionic strength during cleavage and was not caused by intermolecular disulfide bonding.

While high salt aided in ZG16 purification, it did not sufficiently increase protein solubility to permit preparation of a concentrated stock solution for X‐ray crystallography. Reasoning that ZG16 has many exposed aromatic groups, we added arginine to the solution to compete with intermolecular cation‐π interactions. Indeed, ZG16 was much more soluble in the presence of arginine and could be concentrated to at least 12 mg·mL^−1^ (0.75 mm). Crystals were obtained in multiple space groups, all yielding high‐resolution diffraction data (Table 1). Phasing was done by molecular replacement using the known ZG16 core lectin domain structure (PDB; 3APA). Hereafter, ‘crystal form 1’ or ‘structure 1’ will refer to space group P321, at 1.1 Å resolution, and ‘crystal form 2’ or ‘structure 2’ to space group P2_1_2_1_2_1_, at 1.5 Å resolution. Both of these crystals had one ZG16 molecule per asymmetric unit. A second P2_1_2_1_2_1_ crystal was also obtained, diffracting to 1.2 Å resolution and containing two ZG16 molecules per asymmetric unit. This form will be called ‘crystal form 3’ or ‘structure 3’.

**Table 1:** Data collection and refinement statistics. Statistics for the highest‐resolution shell are shown in parentheses.

	Crystal 1	Crystal 2	Crystal 3
Wavelength	0.9724 Å	0.9724 Å	0.9724 Å
Resolution range	43.10–1.08 (1.12–1.08)	32.34–1.50 (1.55–1.50)	33.29–1.20 (1.24–1.20)
Space group	P 32 2 1	P 2_1_ 2_1_ 2_1_	P 2_1_ 2_1_ 2_1_
Unit cell dimensions			
*a* *b* *c* (Å)	58.34 58.34 82.62	38.93 57.00 58.12	44.98 45.42 146.77
α β γ (°)	90 90 120	90 90 90	90 90 90
Total reflections	212 302 (20 830)	76 356 (7146)	340 965 (31 220)
Unique reflections	69 590 (6920)	20 437 (2016)	92 432 (8866)
Multiplicity	3.1 (3.0)	3.7 (3.5)	3.7 (3.5)
Completeness (%)	99.0 (99.6)	97.0 (97.3)	97.3 (95.0)
Mean *I*/sigma (*I*)	11.6 (0.95)	7.9 (0.98)	8.0 (1.04)
Wilson *B*‐factor	13.7	14.0	13.4
*R*‐merge	0.040 (1.00)	0.105 (0.55)	0.082 (0.92)
*R*‐meas	0.048 (1.21)	0.121 (0.65)	0.0948 (1.08)
*R*‐pim	0.026 (0.66)	0.059 (0.32)	0.045 (0.54)
CC1/2	0.999 (0.411)	0.992 (0.760)	0.998 (0.503)
CC*	1 (0.764)	0.998 (0.929)	0.999 (0.818)
Reflections used in refinement	69 584 (6922)	20 343 (2016)	92 419 (8866)
Reflections used for R‐free	1776 (178)	1173 (116)	977 (93)
*R*‐work	0.165 (0.359)	0.171 (0.265)	0.173 (0.321)
*R*‐free	0.175 (0.392)	0.206 (0.312)	0.192 (0.334)
CC (work)	0.974 (0.605)	0.974 (0.842)	0.969 (0.744)
CC (free)	0.968 (0.554)	0.961 (0.859)	0.899 (0.719)
Number of nonhydrogen atoms	1460	1399	2884
Macromolecules	1223	1153	2376
Ligands	7	8	12
Solvent	230	238	496
Protein residues	141	143	286
RMS (bonds)	0.007	0.008	0.008
RMS (angles)	0.97	0.97	1.01
Ramachandran favored (%)	98.55	97.87	97.50
Ramachandran allowed (%)	1.45	2.13	2.50
Ramachandran outliers (%)	0.00	0.00	0.00
Rotamer outliers (%)	0.78	0.00	0.00
Clashscore	0.81	3.01	2.10
Average *B*‐factor	18.3	18.4	18.6
Macromolecules	15.6	16.0	15.9
Ligands	16.4	20.0	15.6
Solvent	32.4	30.2	32.0

### Structures of ZG16 reveal the flexibility of the CXXC region

As described previously, ZG16 has a β‐prism fold with a core composed of three β‐hairpins [21] (Fig. 1B). Following each of the first two β‐hairpins, the polypeptide chain completes a Greek key motif by forming two outer strands hydrogen bonded to either side of the β‐hairpin. Lacking downstream strands, the third β‐hairpin is flanked by ˜ 25 residues from the amino terminus of the protein.

A comparison of the ZG16 structures from the different crystal forms revealed that the protein is flexible at the carboxy terminus, following Val159. Specifically, the peptide bond preceding Pro161 in structure 2 is in the *cis* isomer (it is *trans* in the other structures), and the side chain of Tyr160 is repositioned, apparently to facilitate crystal contacts (Fig. 1C). Notably, despite its flexibility, Tyr160 shows a similar high level of conservation in ZG16 orthologs as amino acids involved in core folding (Trp157) or likely to have a particular functional role in the protein (Cys167) (Fig. 1D). In none of the crystal forms does the hydroxyl group of Tyr160 make a direct hydrogen bond to another part of the protein, raising the possibility that this tyrosine and other conserved residues in the ZG16 tail are under strong evolutionary selection for intermolecular interactions or modifications.

Due to the flexibility of the ZG16 carboxy terminus, the CXXC motif could not be atomically modeled in any of the crystal forms. However, its location could be detected in one of the molecules in the asymmetric unit of crystal form 3. For this molecule, electron density likely corresponding to the guanidinium group of Arg166 was seen adjacent to Asp46, anchoring the rest of the tail (Fig. 1E). Nevertheless, the intervening electron density was not of sufficient quality to unambiguously determine the position of the disulfide. Crystallization of single‐cysteine mutants or wild‐type ZG16 in the presence of reducing agents did not result in improved electron density (data not shown), and it is evident that the carboxy‐terminal segment is highly flexible relative to the ZG16 core lectin domain.

### ZG16 has a dynamic nonproline *cis* peptide bond

**Fig. 2:**
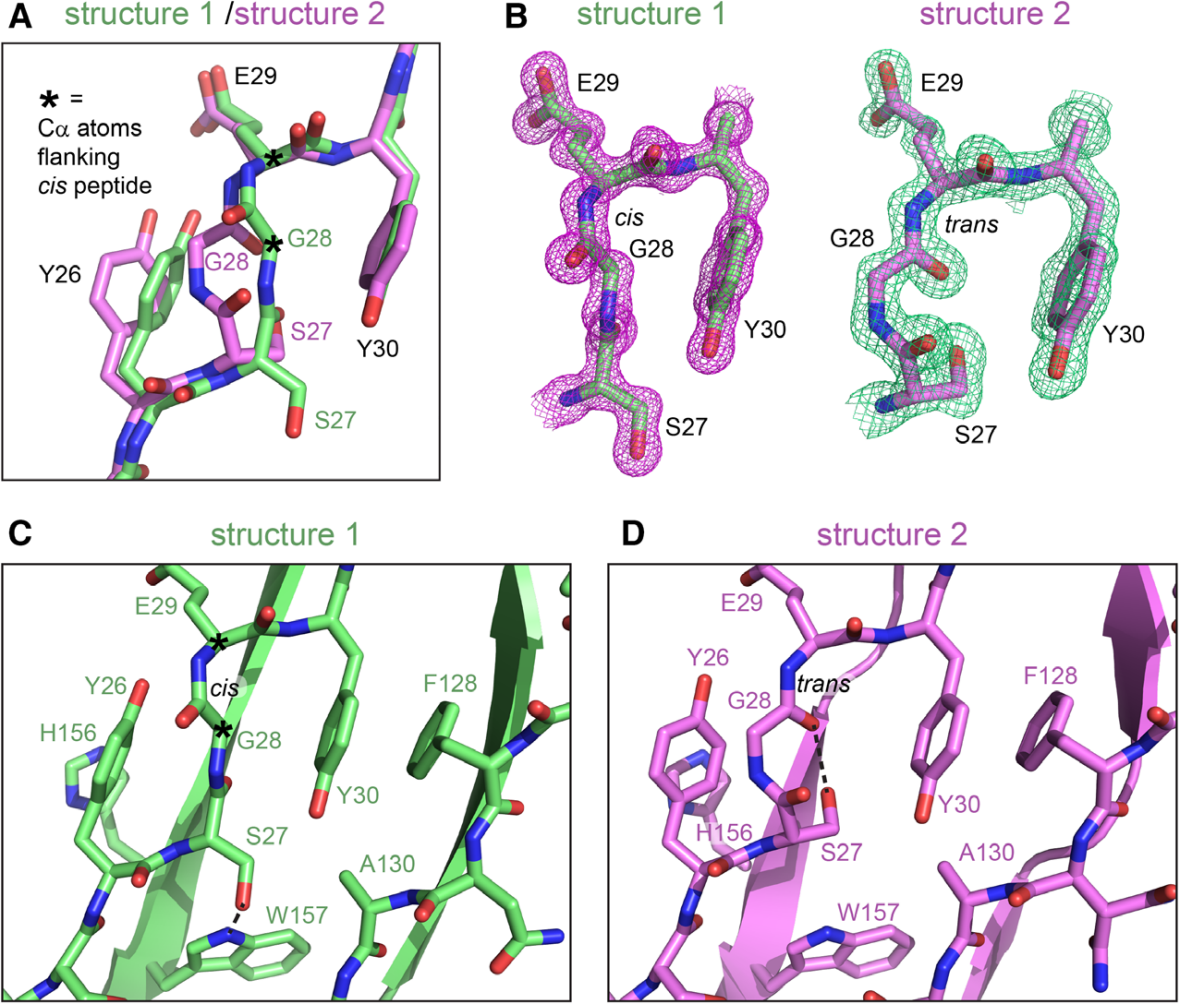
*Cis* and *trans* versions of a non‐Pro peptide bond in ZG16. (A) Superposition of structures 1 and 2. Asterisks indicate the two Cα atoms on either side of the *cis* peptide bond in structure 1. A shift of the Tyr26 side chain accompanies the isomerization of the Gly28‐Glu29 peptide bond. (B) 2Fo‐Fc electron density maps are shown at 2.0σ (structure 1) and 1.5σ (structure 2). (C) Ser27 makes a hydrogen bond to W157 when the Gly28‐Glu29 peptide bond is in *cis*. (D) Ser27 points inwards when the peptide bond is in *trans* and makes hydrogen bonds both to the backbone carbonyl of Gly28 (dashed line) and to the backbone NH of Leu155 in the neighboring strand (not shown). Figure was made using pymol.

In addition to the orientation of the carboxy‐terminal tail, another difference between the ZG16 structures from different crystal forms is the presence or absence of a nonproline (non‐Pro) *cis* peptide bond. A *cis* peptide was observed between Gly28 and Glu29 in structures 1 and 3 (Fig. 2A) and was also present in previous structures of the ZG16 lectin domain (e.g., PDB: 3APA and 3VY7). A non‐Pro *cis* peptide has been noted in a similar position in other β‐prism lectins [22]. The new observation presented here is that the peptide assumes the *trans* configuration in structure 2 (Fig. 2A). These isomers are unambiguous in the high‐quality electron density maps (Fig. 2B). Due to the isomerization, the Cα atoms of Gly28 deviate by 2.5 Å in a superposition of structures 1 and 2, compared to a Cα rmsd of 0.59 Å for residues 22–159. The side chain of Ser27 hydrogen bonds to the backbone N‐H of Leu155 in the final β‐hairpin of structure 2, whereas the Ser27 backbone carbonyl performs this task in structure 1, as in conventional hydrogen bonding between β‐strands. In the structures with the *cis* peptide, the two aromatic rings of Tyr26 and Tyr30 sandwich the Ser27‐Gly28 peptide plane such that the two rings and the peptide are all roughly parallel, while the Tyr26 ring is almost perpendicular to His156 on its opposite side (Fig. 2C). In the *trans* structure, the Ser27‐Gly28 peptide is perpendicular to Tyr26, and the Tyr26 side chain changes conformation such that it lies parallel with His156 (Fig. 2D). The conformational changes upon *cis*‐*trans* isomerization thus propagate to nearby side chains, but the perturbation is resolved within about 6 Å to either side of the isomerizing bond.

### Alternate backbone conformations in a glycan‐binding loop

**Fig. 3:**
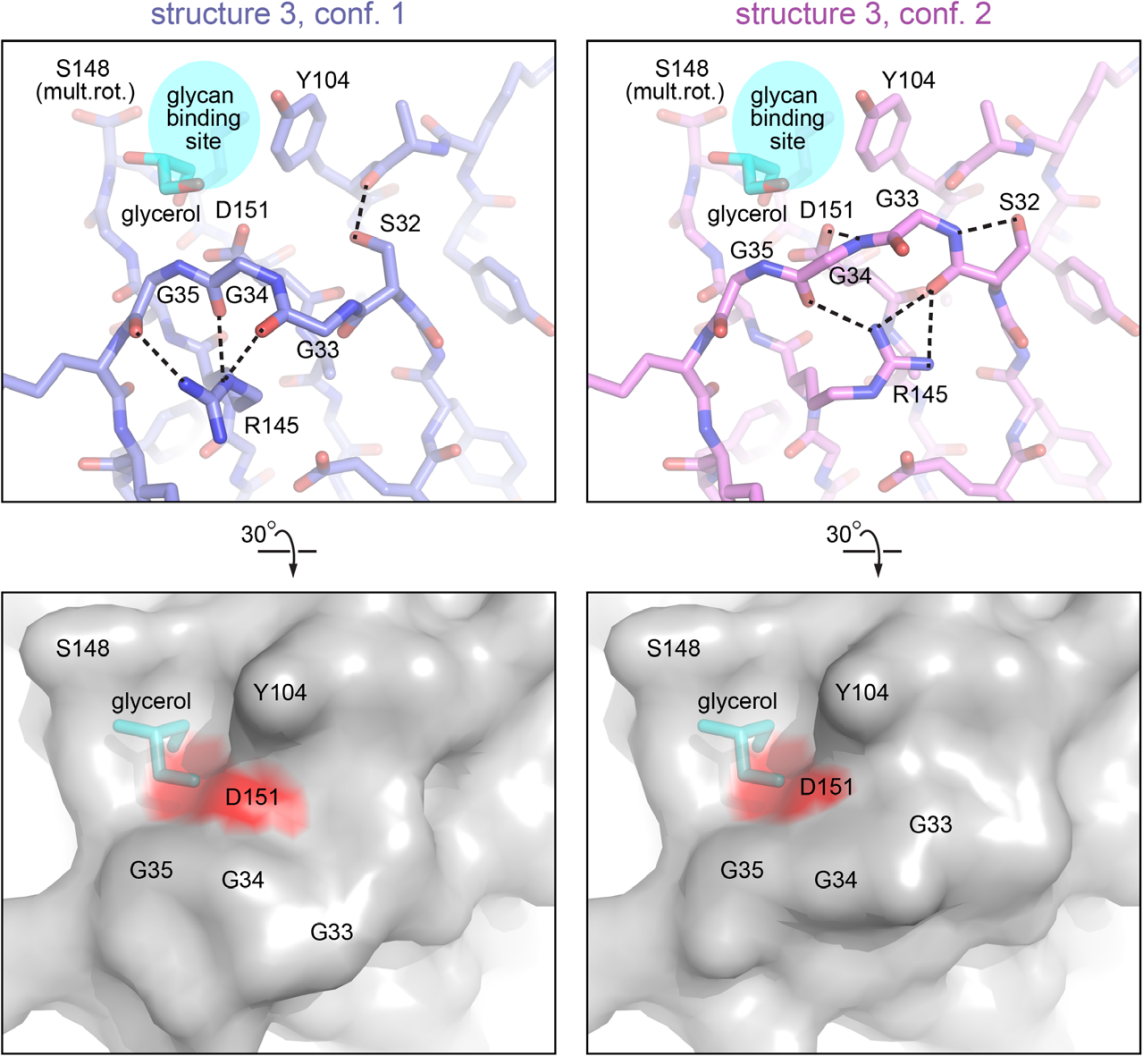
Alternate backbone configurations near the glycan‐binding site of ZG16. Alternate conformations within the same asymmetric unit of crystal form 3 are shown side‐by‐side. A glycerol molecule is bound within the glycan‐binding site, as seen in a previous ZG16 structure [21]. Dashed lines indicate hydrogen bonds. The side chain of Ser148 was observed in multiple rotamers (mult.rot). Bottom panels are rotated 30° relative to the top panels and show the molecular surfaces of the two conformations. The side‐chain oxygen atoms of Asp151 are colored red. Figure was made using pymol.

In addition to the *cis* and *trans* peptide isomers observed in different crystal forms, multiple, alternative backbone conformations superposed in crystallographic electron density were another indication of ZG16 conformational switches. In chain A of structure 3, alternate conformations were detected in the region from Ser32 to Gly34 (Fig. 3), corresponding to the glycine‐rich ‘GG loop’ (Fig. 1B), one of the main elements of the glycan‐recognition site [15, 16]. The high‐resolution diffraction data facilitated interpretation of this switch, in which movement of the GG loop main chain is coupled to rotamer changes in the side chain of Arg145 (Fig. 3). The effect of the conformational changes in this loop is to modulate the contours of the glycan‐binding pocket, re‐configure the constellation of hydrogen bond donors and acceptors, and alter the accessibility of Asp151 (Fig. 3), a conserved amino acid important for mannose recognition, adhesion to pathogenic fungi, and anti‐cell proliferative activities observed for ZG16 [3, 23].

### The ZG16 CXXC motif is oxidized *in vitro* by PDI and QSOX1

**Fig. 4:**
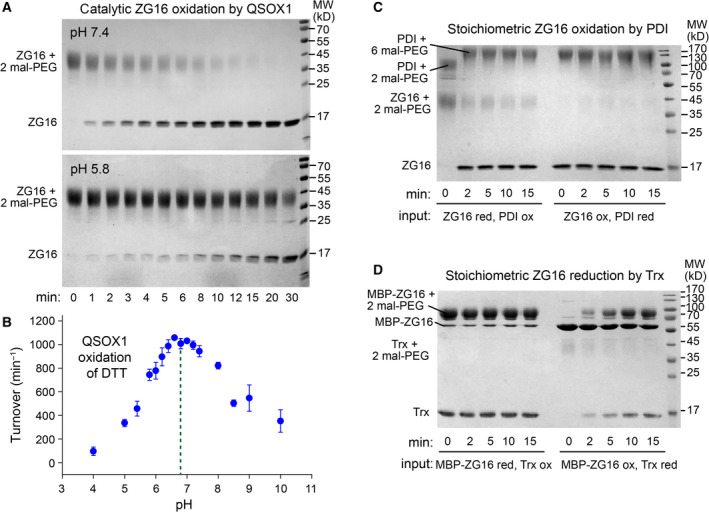
Oxidation of ZG16 by QSOX1 and PDI and reduction by Trx. (A) 50 nm QSOX1 was supplied to 10 µm reduced ZG16 in buffers at the indicated pH values. Aliquots were taken and the reaction quenched by addition of mal‐PEG. Gel shifts corresponding to 10–15 kD are typically seen for each modification with mal‐PEG 5 kD [43], so the observed shift is consistent with modification of the two cysteines in reduced ZG16. No appreciable oxidation of ZG16 occurs over this time period in the absence of QSOX1 (not shown). (B) QSOX1 turnover on DTT measured as a function of pH. Error bars are standard deviations of three measurements. The dashed vertical line shows the approximate midpoint of the peak of activity. (C) Reduced ZG16 (ZG16 red) (5 µm) was incubated with oxidized PDI (PDI ox) (2.5 µm) or *vice versa* (ZG16 ox, PDI red) for the indicated times. Reactions were quenched by addition of mal‐PEG. Human PDI has two unpaired cysteines that become modified with mal‐PEG regardless of the redox state of the active sites. (D) Reduced ZG16 fused to MBP (MBP‐ZG16 red) (4 µm) was incubated with oxidized Trx (Trx ox) (4 µm) or *vice versa* (MBP‐ZG16 ox, Trx red) for the indicated times. Reactions were quenched by addition of mal‐PEG. The MBP‐ZG16 fusion protein was used in this experiment so that ZG16 and Trx could be clearly distinguished on the gel.

The enzyme quiescin sulfhydryl oxidase 1 (QSOX1) is a catalyst of disulfide bond formation localized, like ZG16, to the Golgi apparatus and extracellular fluids [24, 25]. Moreover, QSOX1 is highly expressed in goblet cells of the colon [26] and is present with ZG16 in the mucus‐associated proteome [27]. We therefore asked whether the ZG16 CXXC motif is a substrate of QSOX1. Reduced ZG16 was prepared and incubated with catalytic amounts of recombinant human QSOX1 at either neutral or moderately acidic pH, the latter relevant to the Golgi. Time points were taken by removing aliquots and quenching with maleimide‐functionalized polyethylene glycol (mal‐PEG) of molecular mass 5 kD. At pH 7.4, QSOX1 efficiently catalyzed formation of a disulfide in ZG16, as measured by loss of ZG16 reactivity with mal‐PEG following addition of QSOX1 (Fig. 4A). At pH 5.8, the reaction was slower (Fig. 4A), consistent with the pH profile of human QSOX1 activity measured on the small‐molecule model substrate dithiothreitol (DTT) (Fig. 4B) and with that previously reported for *Trypanosoma brucei* QSOX1 [28]. Due to the limitations of ZG16 solubility in physiologically relevant solutions, the Michaelis constant (*K*_M_) could not be established, but we estimate it to be above 150 µm based on oxygen consumption assays (data not shown).

We also tested whether reduced ZG16 can be oxidized by protein disulfide isomerase (PDI), a major oxidoreductase involved in disulfide bond formation in the ER [29] and secreted in certain physiological settings [30]. As PDI does not perform multiple turnovers without an additional electron sink, we supplied it to reduced ZG16 in a 1 : 2 stoichiometry, considering that PDI has two active‐site CXXC motifs. PDI rapidly oxidized ZG16 in this experiment (Fig. 4C). In contrast, *E. coli* Trx reduced the ZG16 disulfide (Fig. 4D), indicating that the redox potential of the ZG16 CXXC motif is between those of PDI (˜ 165 mV) [31] and Trx (−270 mV) [32].

## Discussion

ZG16 is an animal lectin with many reported disease associations [4, 5, 6, 7, 8, 9] but a poorly understood physiological function. One aspect of ZG16 that had not been addressed is the nature of its carboxy‐terminal cysteines. This study aimed to determine the structural relationship of the ZG16 CXXC motif to the β‐prism fold, as well as to provide an initial analysis of ZG16 redox properties. We first and foremost observed that the protein has little tendency to form disulfide bonded dimers or multimers. Instead, the two cysteines in the human ZG16 tail readily formed an intramolecular disulfide, both when purified from bacterial cell lysates (starting material in Fig. 4C) and when oxidized by QSOX1 (Fig. 4A). We found ZG16 to be an excellent substrate for QSOX1 *in vitro* (Fig. 4A) and thus potentially useful for sulfhydryl oxidase assays in the future. ZG16 may also be a physiological substrate of QSOX1, since these proteins are found together in goblet cells and in intestinal secretions [12, 27]. Nevertheless, it should not be concluded on the basis of our *in vitro* experiments that QSOX1 necessarily oxidizes ZG16 *in vivo*. We have shown that PDI can also oxidize ZG16 (Fig. 4C), and it may do so in the ER prior to any encounter between ZG16 and QSOX1. Though ZG16 is oxidized *in vitro* by QSOX1 and PDI, it is reduced by *E. coli* Trx (Fig. 4D). Whether ZG16 would encounter Trx from intestinal bacteria is unknown, but human Trx, which has a redox potential of −230 mV [33], has been identified in mucus [34]. The roles of various redox‐active proteins in the intestinal mucus layer is an intriguing but still largely unexplored topic.

When contemplating a possible redox‐related function for ZG16, an evolutionary perspective may add insight. Overall, the ZG16 amino acid sequence is highly conserved in the animal species that contain the protein. Birds appear to have lost ZG16 altogether, but a ZG16 coding sequence has been identified in some reptiles, amphibians, and in many mammals. Most ZG16 orthologs contain the CXXC motif, but Cetartiodactyla (whales and even‐toed ungulates) and some other species lack the first cysteine. The lower conservation of the first cysteine can be seen in Fig. 1D. Notably, Tyr160 and Pro161, which are present in the flexible region following the last β‐strand of the ZG16 fold (Fig. 1C), are conserved both in ZG16 variants with a CXXC and in those with a single cysteine. As noted above, Tyr160 does not make hydrogen bonds or fixed hydrophobic contacts within ZG16, so this residue may be conserved for functional intermolecular interactions. Presuming that ZG16 uses its carboxy‐terminal tail for such interactions, co‐evolution of a ZG16 partner may explain how a single cysteine could substitute for a disulfide bond in some species. The single‐cysteine ZG16 variants have a histidine or tyrosine in place of the missing cysteine, followed almost always by a glutamic acid. This glutamic acid is not found in orthologs with both cysteines. The distinct evolutionary bifurcation of the ZG16 carboxy‐terminal tail, as well as the apparent absence of the protein in birds (which have evolved unique aspects of their digestive systems [35]), may provide hints to the physiological role of ZG16.

One open question relating to the structure and function of ZG16 is the relationship between its redox and glycan‐binding activities. A previous study noted that ZG16 did not show antimicrobial activity and that reduction of the disulfide did not activate such activity [14]. Based on our data, the CXXC tail appears to be structurally uncoupled from the glycan‐binding region. Many sugar‐binding studies of ZG16 were performed in the absence of the tail entirely [15, 16]. The lack of a requirement for the CXXC motif in glycan binding is expected based on the presence of this motif on the opposite end of the β‐prism (Fig. 1B) and its conformational heterogeneity. Nevertheless, interactions made by the ZG16 tail may indirectly affect glycan binding *in vivo* by controlling localization of the protein and contributing to avidity.

Other conformational changes we observed in ZG16 may, in contrast, be directly linked to glycan binding and may help explain the diversity of binding targets. Our set of ZG16 crystal structures revealed two main regions of flexibility near the glycan‐binding site. One is the first strand of the first β‐hairpin, and the second is the GG loop following this first strand. The first strand is the site of *cis‐trans* isomerization of the Gly28‐Glu29 peptide bond. The part of ZG16 affected by this isomerization is adjacent to the region that shows NMR chemical shift differences upon binding of phosphatidylinositol mannosides and overlaps the region that participates in heparin binding [15, 16]. The GG loop, in turn, interacts with bound mannose derivatives [16]. It was previously shown that sugars can bind ZG16 in different orientations [15], but our work raises the possibility of another mechanism for recognition of diverse glycans: structural reorganization of the glycan‐binding pocket.

It has been noted that non‐Pro *cis* peptide bonds are detected most frequently in proteins involved in sugar binding and catalysis and that these isomers are usually functionally important [36]. Nevertheless, experimental observation of both the *cis* and *trans* isomers of the same protein is rare. Whether isomerization contributes to the activity of ZG16 remains to be determined. Importantly, though the Gly28‐Glu29 region of structure 2 (bearing a *trans* peptide bond) is near a crystal contact, introducing the *cis* peptide configuration as observed in the other ZG16 structures shows that it would not have induced a steric clash at this contact. Neither do any particularly noteworthy intermolecular interactions seem to be enabled uniquely by the *trans* form. Thus, it does not appear that crystal packing in crystal form two stabilized a high‐energy conformation of ZG16 either to avoid unfavorable interactions or to facilitate favorable ones. For reference, another amino acid participating in the same protein–protein interface, Leu133, clearly occupies a different rotamer in structure 2 than in the other structures to avoid a clash. Leu133 is about 10 Å away from Gly28, and the Leu133 rotamer is not coupled in any obvious way to the isomerizing bond. It would be interesting to determine whether physiological interactions of ZG16 with partner or target proteins stabilize the *trans* form of the Gly28‐Glu29 peptide and for what functional purpose. Overall, the set of structures and redox activity assays described here emphasize the malleability of the ZG16 glycan‐binding region and show that ZG16 can engage in diverse dithiol‐disulfide exchange reactions.

## Materials and methods

### Protein production and purification

The coding sequence for residues 21–167 of ZG16 was inserted into a plasmid downstream of the coding sequences for MBP, a His_6_ tag, and a TEV protease cleavage site. The signal peptide for entry of ZG16 into the secretory pathway, predicted to span residues 1 through 16 [37], was not necessary for expression in *E. coli*, and the starting residue was chosen based on previous work [17]. According to the plasmid design, a non‐native glycine remained at the amino‐terminus of ZG16 after TEV cleavage.

The ZG16 expression plasmid was transformed into the BL21(DE3) *E. coli* strain. Cultures were grown at 37 °C in the presence of 100 mg·L^−1^ ampicillin to an optical density of 0.5 at 595 nm, at which point isopropyl β‐d‐1‐thiogalactopyranoside was added to a concentration of 0.5 mm. The growth temperature was lowered to 25 °C, and the cultures were left to shake overnight. Cells were then pelleted, resuspended in 5 mm sodium phosphate, pH 7.5, 400 mm NaCl, and 5 mm imidazole (cell suspension buffer), and frozen at −80 °C.

To purify ZG16, cell suspensions were thawed, sonicated on ice, and spun at 25 000 ***g*** for 20 min at 4 °C. Supernatant was applied to a Ni‐NTA column, washed in the cell suspension buffer, and then eluted with an increasing imidazole gradient. Eluted protein was diluted threefold with PBS supplemented with an additional 800 mm NaCl, placed into a 3 kDa cutoff dialysis bag together with His_6_‐tagged TEV protease, and dialyzed against the high‐salt PBS overnight at room temperature. High salt prevented protein precipitation at this step. The cleaved protein was then reapplied to a Ni‐NTA column equilibrated in 50 mm sodium phosphate buffer, pH 7.5, and 500 mm NaCl. ZG16 was released from the column during a wash with cell suspension buffer, and the His_6_‐tagged MBP and TEV protease were released during a gradient to higher imidazole concentrations. ZG16 protein concentration was measured by absorbance in 6 m guanidine, 20 mm sodium phosphate buffer, pH 6.8, using an extinction coefficient of 31 500 m ^−1^·cm^−1^.

Human PDI (NP_000909.2) was cloned into the pcDNA3.1 plasmid to produce the full‐length protein with a His_6_ tag following the KDEL sequence at the carboxy terminus. The PDI expression vector was transfected using the PEI Max reagent (Polysciences Inc., Warrington, PA, USA) into HEK 293F cells (Thermo Fisher, Waltham, MA, USA) grown in FreeStyle 293 expression medium. Six days after transfection, the culture was harvested and subjected to centrifugation for 15 min at 500 ***g*** to remove cells. The supernatant was transferred to a fresh bottle and centrifuged for 15 min at 2000 ***g*** to remove particulate matter. The remaining supernatant was passed through a 0.45 µm filter, and protein was purified by Ni‐NTA chromatography. The catalytic region of human QSOX1 was purified in a similar manner using an expression vector previously described [38]. *Escherichia coli* Trx was purified from bacteria essentially as described except without detergent [39].

### Crystallization and structure solution

For crystallization, ZG16 was concentrated to 12 mg·mL^−1^. To aid in solubility during this process, 200 mm l‐arginine was added to the protein in the centrifugal concentrator, and the buffer was exchanged to 10 mm Tris, pH 8.1, 100 mm NaCl, and 200 mm l‐arginine by repeated concentration and dilution.

Crystals were grown using the hanging drop method by mixing protein stock solution 1 : 1 with well solution. Block‐like crystals (crystal form 1) were obtained from the JCSG‐plus™ screen (Molecular Dimensions, Sheffield, UK) in 0.1 m 4‐(2‐hydroxyethyl)‐1‐piperazineethanesulfonic acid buffer (HEPES), pH 7.0, 30% v/v Jeffamine® ED‐2003 (Molecular Dimensions). Rod‐shaped crystals (crystal form 2) were grown in 0.2 m sodium thiocyanate, 20% w/v polyethylene glycol 3350. Another rod‐shaped crystal (crystal form 3) was grown in 1.0 m ammonium sulfate, 0.1 m BIS‐Tris, pH 5.5, 1% w/v polyethylene glycol 3350. In all cases, crystals were transferred to a solution composed of 80% of the well solution and 20% glycerol before flash freezing. Diffraction data were collected at the European Synchrotron Radiation Facility on beamline ID23‐1.

Phases were obtained by molecular replacement using PDB: 3APA as the search model. Rebuilding and refinement were conducted iteratively using coot [40] and Phenix [41]. Structure figures were generated using pymol [42]. Atomic coordinate files have been deposited in the Protein Data Bank with accession codes 7O4P (structure 1), 7O3I (structure 2), and 7O88 (structure 3).

### Oxidation assays

Reduced ZG16 was prepared by adding DTT at a concentration of 20 mm to a 200 µL aliquot of 100 µm protein. After 20 min at room temperature, DTT was removed from the protein solution using a PD‐10 column equilibrated in PBS supplemented with an additional 400 mm NaCl. Elution fractions of 500 µL were collected, and the protein concentration in the peak fraction was measured. Mal‐PEG of molecular mass 5 kDa was dissolved in water at a concentration of 50 mm and applied to a PD‐10 column equilibrated in water to remove any maleimide not conjugated to polyethylene glycol. Reactions were initiated containing 10 µm reduced ZG16 and 50 nm recombinant human QSOX1. At the indicated time points, 9 µL aliquots of the reaction were removed and mixed with 1 µL of ˜ 10 mm mal‐PEG. For dithiol‐disulfide exchange reactions with PDI, PDI (40 µm) was reduced by incubation with 20 mm DTT for 20 min at room temperature. Reduced PDI was desalted on a PD‐10 column equilibrated with PBS. Oxidized PDI and reduced ZG16, or *vice versa*, were mixed at final concentrations of 5 µm ZG16 and 2.5 µm PDI in 10 µL aliquots. At the indicated time points, 1 µL mal‐polyethylene glycol was added to each aliquot. Gel loading buffer was added to the aliquots, and proteins were separated on 12% acrylamide gels.

The pH dependence of human QSOX1 was measured by monitoring oxygen consumption coupled to DTT oxidation in an Oxygraph Clark‐type oxygen electrode. Reactions of 1 mL contained 100 nm QSOX1 and 1 mm DTT in 50 mm buffer, 65 mm NaCl, and 1 mm EDTA. Buffers were sodium acetate (pH 4.0, 5.0, and 5.5), sodium phosphate (pH 5.8, 6.0, 6.2, 6.4, 6.6 6.8, 7.0, 7.2, 7.4, and 8.0), Tris (pH 8.5 and 9.0) and *N*‐cyclohexyl‐2‐aminoethanesulfonic acid (CHES; pH 10.0).

## Conflict of interest

The authors declare no conflict of interest.

## Author contributions

DF planned experiments, and all authors performed experiments and analyzed data. DF wrote the paper with assistance from all other authors.

### Peer Review

The peer review history for this article is available at https://publons.com/publon/10.1111/febs.16044.

## Data Availability

The structural data that support these findings are openly available in the wwPDB at https://doi.org/10.2210/pdb7O4P/pdb, https://doi.org/10.2210/pdb7O3I/pdb, https://doi.org/10.2210/pdb7O88/pdb.
